# Keratinocyte Growth Factor Induces Gene Expression Signature Associated with Suppression of Malignant Phenotype of Cutaneous Squamous Carcinoma Cells

**DOI:** 10.1371/journal.pone.0033041

**Published:** 2012-03-12

**Authors:** Mervi Toriseva, Risto Ala-aho, Sirkku Peltonen, Juha Peltonen, Reidar Grénman, Veli-Matti Kähäri

**Affiliations:** 1 Department of Dermatology, University of Turku, Turku University Hospital, Turku, Finland; 2 MediCity Research Laboratory, University of Turku, Turku, Finland; 3 Turku Graduate School of Biomedical Sciences, Turku, Finland; 4 Department of Cell Biology and Anatomy, University of Turku, Turku, Finland; 5 Department of Otorhinolaryngology-Head and Neck Surgery, Turku University Hospital, Turku, Finland; University Hospital Hamburg-Eppendorf, Germany

## Abstract

Keratinocyte growth factor (KGF, fibroblast growth factor-7) is a fibroblast-derived mitogen, which stimulates proliferation of epithelial cells. The expression of KGF by dermal fibroblasts is induced following injury and it promotes wound repair. However, the role of KGF in cutaneous carcinogenesis and cancer progression is not known. We have examined the role of KGF in progression of squamous cell carcinoma (SCC) of the skin. The expression of KGF receptor (KGFR) mRNA was lower in cutaneous SCCs (n = 6) than in normal skin samples (n = 6). Expression of KGFR mRNA was detected in 6 out of 8 cutaneous SCC cell lines and the levels were downregulated by 24-h treatment with KGF. KGF did not stimulate SCC cell proliferation, but it reduced invasion of SCC cells through collagen. Gene expression profiling of three cutaneous SCC cell lines treated with KGF for 24 h revealed a specific gene expression signature characterized by upregulation of a set of genes specifically downregulated in SCC cells compared to normal epidermal keratinocytes, including genes with tumor suppressing properties (*SPRY4*, *DUSP4*, *DUSP6*, *LRIG1*, *PHLDA1*). KGF also induced downregulation of a set of genes specifically upregulated in SCC cells compared to normal keratinocytes, including genes associated with tumor progression (*MMP13*, *MATN2*, *CXCL10*, and *IGFBP3*). Downregulation of MMP-13 and KGFR expression in SCC cells and HaCaT cells was mediated via ERK1/2. Activation of ERK1/2 in HaCaT cells and tumorigenic Ha-*ras*-transformed HaCaT cells resulted in downregulation of MMP-13 and KGFR expression. These results provide evidence, that KGF does not promote progression of cutaneous SCC, but rather suppresses the malignant phenotype of cutaneous SCC cells by regulating the expression of several genes differentially expressed in SCC cells, as compared to normal keratinocytes.

## Introduction

Keratinocyte growth factor (KGF, fibroblast growth factor-7 (FGF-7)) is produced by cells of mesenchymal origin and by epidermal γδ T cells [1], [2]. KGF binds to a specific cell surface receptor (KGFR), the splicing variant IIIb of FGF-receptor-2 (FGFR2-IIIb), which is expressed exclusively by various types of epithelial cells [3]. KGF is mitogenic for epithelial cells and it regulates epidermal morphogenesis, differentiation and homeostasis [1], [4]–[6]. In normal wound healing, the expression of KGF by stromal fibroblasts is induced after injury in response to various stimuli including transforming growth factor-α, interleukin-1, and tumor necrosis factor-α [7]–[9]. The pivotal role of KGFR signaling in wound repair was demonstrated by showing that expression of dominant-negative form of KGFR in mouse epidermis results in delayed wound closure [6]. In addition, the stimulatory effect of KGF on cutaneous wound healing has been demonstrated by delivery of exogenous KGF into wounds [10]–[12]. Recently, KGF has also been recognized for its protective effect on normal epithelial tissues, and use of recombinant KGF has been approved for prevention and treatment of severe oral mucositis in patients with hematologic cancers receiving high-dose chemotherapy and radiation therapy followed by bone marrow transplantation. However, the safety and efficacy of KGF for protecting normal epithelial tissue in patients with non-hematologic malignancies treated with chemo- and radiotherapy has not been established and is under investigation [13]–[15].

Cutaneous squamous cell carcinoma (SCC) is the second most common malignant tumor of skin, and its incidence is increasing globally [16]. Chronic ulceration is a well recognized risk factor for cutaneous SCC and ulceration is a typical clinical feature during progression of UV-induced cutaneous SCC from intraepithelial early lesion (actinic keratosis) to invasive and metastatic SCC [17]. Therefore, many characteristics of normal wound healing, including proliferation of epidermal keratinocytes, inflammation, and angiogenesis, are also typical features in cutaneous SCCs. The role of KGF in malignant transformation of epithelial cells and in progression of epithelial cancers has been studied with variable findings. The expression of KGF and KGFR correlates with venous invasion of pancreatic cancer [18]. KGF and KGFR expression is high in poorly differentiated lung adenocarcinoma and in well differentiated lung SCC [19]. In esophageal cancer KGFR expression correlates with differentiation, but the expression of KGF correlates with lymphatic invasion [20]. KGFR expression is also associated with tumor suppression in bladder carcinoma and prostate cancer [21]–[23]. Interestingly, proliferation of human head and neck SCC cell lines is not stimulated by KGF [24]. KGF had no effect on the efficacy of chemotherapy on KGFR positive head and neck SCC and colorectal cancer xenografts [13]. In contrast, KGF was shown to counteract anticancer treatment of breast cancer cell lines [15]. Thus, depending on cancer type, KGF may exert tumor promoting or suppressing effects or may not have any effect on cancer cell behavior [13], [15], [23], [24]. However, at present, the role of KGF in the progression of cutaneous SCC is not known.

In the present study, we have examined the effect of KGF on cutaneous SCC cells. The results show that although most SCC cell lines express KGFR, KGF does not stimulate their proliferation. Furthermore, the expression level of KGFR mRNA correlates negatively with tumorigenic potential of Ha-*ras*-transformed HaCaT cells. KGF reduces invasion capacity of KGFR-positive cutaneous SCC cells and induces a specific gene expression signature characterized by regulation of the expression of several genes differentially expressed in SCC cells, as compared to normal epidermal keratinocytes. Based on these findings, we propose that KGF does not promote progression of cutaneous SCCs, but rather suppresses the malignant phenotype of SCC cells.

## Results

### Expression of KGFR and KGF by cutaneous SCC tumor cells

**Figure 1:**
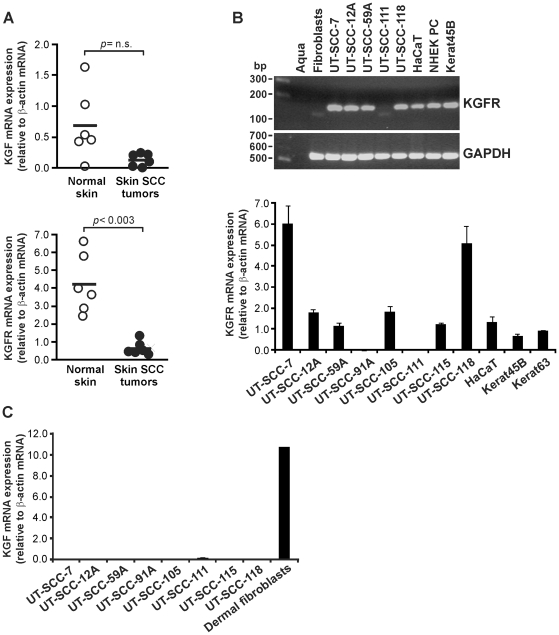
Expression of KGF and KGFR mRNA in cutaneous SCC tumors and cell lines. (**A**) Cutaneous SCC tumor tissue samples (n = 6) and normal skin samples (n = 6) were analyzed for KGF (upper panel) and KGFR (lower panel) mRNA levels by qPCR. The results were normalized for β-actin mRNA levels in each sample. A dot represents the mean of duplicate analysis of one sample. Statistical analysis with independent samples T-test (n.s., not significant). (**B**, upper panel) Human skin SCC cell lines (UT-SCC-7, -12A, -59A, -111, -118), HaCaT cells and normal epidermal keratinocytes (NHEK PC, Kerat45B) were analyzed for the expression of KGFR mRNA using RT-PCR. RNA from normal human skin fibroblasts served as a negative control. Amplification of a fragment of housekeeping gene GAPDH transcript was used as a loading control. (**B**, lower panel) KGFR mRNA expression was quantified in human skin SCC cell lines (UT-SCC-7, -12A, -59A, -91A, -105, -111, -115, and -118), HaCaT cells and normal epidermal keratinocytes (Kerat45B, Kerat63) with qPCR (n = 3–4). (**C**) KGF mRNA expression was quantified in human skin SCC cell lines (UT-SCC-7, -12A, -59A, -91A, -105, -111, -115, and -118), and normal human dermal fibroblasts with qPCR (n = 3–4).

In order to elucidate the role of KGF in progression of epidermal malignant tumors, we first determined the expression of KGF and KGFR mRNA in cutaneous SCCs and in normal skin by quantitative real-time RT-PCR (qPCR). The expression of KGF mRNA in cutaneous SCCs (n = 6) was detectable and comparable to that in normal skin (n = 6) (Figure 1A, upper panel). The mean level of KGFR transcript expression was significantly lower in cutaneous SCC tumors than in normal skin (Figure 1A, lower panel).

Next, the expression of KGFR in cutaneous SCC cell lines, normal primary epidermal keratinocytes, and in HaCaT cells, an epidermal keratinocyte-derived non-tumorigenic cell line with p53 inactivation [25] was analyzed by RT-PCR. A specific 150 bp fragment representing the specific IIIb-type exon of FGFR2-receptor transcript was amplified from 4 out of 5 SCC cell lines examined, from HaCaT cells, and from epidermal keratinocytes (Figure 1B, upper panel). As expected, dermal fibroblasts were negative for KGFR transcript.

The expression of KGFR by cutaneous SCC cells was further determined by qPCR. KGFR mRNA was absent in 2 out of 8 cutaneous SCC cell lines and in 4 out of 8 SCC cell lines the level of expression was comparable to normal epidermal keratinocytes and HaCaT cells (Figure 1B, lower panel). Elevated levels of KGFR transcript, as compared to normal keratinocytes, were noted in 2 out of 8 SCC cell lines (Figure 1B, lower panel). KGF mRNA was virtually absent in all 8 skin SCC cell lines examined by qPCR, whereas it was expressed at high level by primary dermal fibroblasts in culture (Figure 1C).

### Lack of mitogenic response to KGF in cutaneous SCC cells

**Figure 2:**
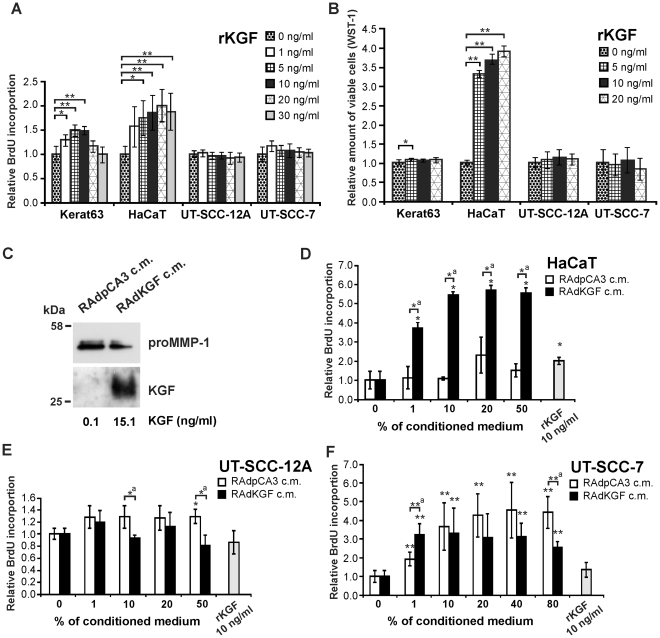
Lack of mitogenic response to KGF in skin SCC cells. (**A**) Growth supplement-starved normal primary keratinocytes (Kerat63), serum-starved HaCaT cells and primary (UT-SCC-12A) and metastatic (UT-SCC-7) cutaneous SCC cells were treated with recombinant KGF (rKGF; 10 ng/ml) for 2 h followed by addition of BrdU. DNA-synthesis was determined as incorporation of BrdU into DNA after 18 h incubation. *p<0.02; **p<0.005, with Mann-Whitney U-test, n = 5–6. (**B**) Keratinocytes (Kerat63) (n = 8), HaCaT cells (n = 12), UT-SCC-12A (n = 12) and UT-SCC-7 (n = 12) cells were treated as in (A). The relative amount of viable cells was determined using colorimetric assay of WST-1 reagent metabolism. *p<0.02; **p<0.000001, with Mann-Whitney U-test. (**C**) Serum-starved primary human skin fibroblasts were infected with recombinant adenovirus (RAdKGF) coding for human KGF, or with empty control adenovirus (RAdpCA3) (MOI 200) overnight and incubated in DMEM containing 0.5% FCS for 72 h. The conditioned medium (c.m.) was analyzed for the presence of KGF by western blotting, and KGF concentration was quantified with ELISA. ProMMP-1 was visualized as control in c.m. by western blotting. (**D–F**) Serum-starved HaCaT cells (**D**), UT-SCC-12A cells (**E**) and UT-SCC-7 (**F**) cells were incubated with c.m. in indicated concentrations or with rKGF for 18 h. BrdU was added and DNA-synthesis was analyzed after 8 h incubation. *^a^ *p*<0.05, **^a^ *p*<0.01, *p<0.05 compared to 0% c.m., **p<0.02 compared to 0% c.m. with Mann-Whitney U-test, n = 4–5.

To further examine the KGF response of cutaneous SCC cells, two KGFR positive SCC cell lines, one primary (UT-SCC-12A) and one metastatic (UT-SCC-7), as well as normal epidermal keratinocytes and HaCaT cells were stimulated with various concentrations of recombinant KGF for 20 h, and BrdU incorporation was determined as a marker for DNA synthesis. As shown in Figure 2A, KGF treatment enhanced DNA synthesis of normal keratinocytes and HaCaT cells, but had no effect on the SCC cell lines tested. Incubation with KGF for 48 h also increased number of viable cells in keratinocyte and HaCaT cell cultures, but not in two SCC cell line cultures (Figure 2B).

To examine the effect of fibroblast produced KGF on SCC cells, normal human skin fibroblasts were transduced with recombinant adenovirus encoding human KGF (RAdKGF), or with empty control adenovirus (RAdpCA3), and cell culture media were harvested 72 h later. High concentration of human KGF was detected in medium of RAdKGF transduced fibroblasts by Western blot analysis and ELISA (Figure 2C). Serum starved SCC cells and HaCaT cells were cultured with various concentrations (1–80%) of conditioned medium from either RAdKGF or RAdpCA3 transduced fibroblasts for 26 h. A concentration-dependent stimulation of DNA synthesis was noted in HaCaT cells cultured with medium from RAdKGF transduced fibroblasts, as compared to conditioned medium of RAdpCA3 transduced dermal fibroblasts (Figure 2D). In contrast, DNA synthesis of SCC cells was not stimulated by medium from RAdKGF-transduced fibroblasts, and was even reduced by highest concentrations of conditioned media (Figure 2E,F).

### KGF induces a specific gene expression signature in cutaneous SCC cells

To further elucidate the effect of KGF on SCC cells, three KGFR-positive cutaneous SCC cell lines were treated with KGF for 24 h. Global gene expression profiling of KGF-treated SCC cells, corresponding untreated control cells and untreated normal keratinocytes was performed by oligonucleotide microarray (Affymetrix). The expression of several genes involved in cell growth, cellular signaling and regulation of cell cycle, as well as extracellular and intracellular maintenance was clearly elevated in KGF-treated skin SCC cell lines (Table 1). Five genes with tumor suppressing properties, *i.e. SPRY4* (Sprouty homolog 4) [26], *DUSP4* and *DUSP6* (dual-specificity phosphatases 4 and 6) [27], [28], *LRIG1* (Leucine-rich repeats and Ig-like domains 1) [29] and *PHLDA1* (Pleckstrin homology-like domain family A, member 1) [30], were upregulated by KGF (1.5–2.1 fold) (Table 1). Altogether 11 genes (including *DUSP4*, *SPRY4*, and *ETV5*), which were specifically downregulated in SCC cell lines (more than 2-fold) compared to normal keratinocytes were upregulated by KGF. In contrast, five genes with elevated expression levels in SCC cell lines compared to normal keratinocytes were further upregulated by KGF (Table 1).

**Table 1:** Upregulation of different classes of genes in cutaneous SCC cells by KGF as determined by DNA microarray analysis.

Coded protein (*Gene name*)	Ctrl	rKGF^1^	SCC/normal^2^
**ECM/ECM receptors, ECM modulators, cytoskeleton**			
Hyaluronan synthase 2 (*HAS2*)	1	2.2	∼
Vimentin (*VIM*)	1	1.6	−3.5
Leupaxin (*LPXN*)	1	1.7	∼
Adducin 2 (beta), transcript variant beta-4 (*ADD2*)	1	1.5	3.7
**Micellaneous**			
Anthrax toxin receptor 2 (*ANTXR2*)	1	2.2	−2.3
Tissue factor pathway inhibitor 2 (*TFPI2*)	1	2.0	−3.4
Lung cancer metastasis-associated protein (MAG1, *AGPAT9*)	1	1.9	∼
Homo sapiens hypothetical LOC390345	1	1.8	1.7
Arginase, type 2 (*AGR2*)	1	1.8	−1.8
5′-nucleotidase, ecto (CD73, *NT5E*)	1	1.8	∼
Ankyrin repeat domain 22 (*ANKRD22*)	1	1.8	−3.4
Myeloma overexpressed (*MYEOV*)	1	1.7	2.1
SH3 domain and tetratricopeptide repeats 2 (*SH3TC2*)	1	1.6	∼
Heparan sulfate glucosamine 3-O-sulfotransferase 1 (*HS3ST1*)	1	1.6	∼
Ornithine decarboxylase 1 (*ODC1*)	1	1.6	−2.9
Discoidin, CUB and LCCL domain containing 2 (*DCBLD2*)	1	1.6	∼
Semaphorin 3A (*SEMA3A*)	1	1.5	3.3
Carboxylesterase 1 (*CES1*)	1	1.5	−2.0
ATP-binding cassette, sub-family A , member 13 (*ABCA13*)	1	1.5	2.6
**Growth factors/ receptors/ signaling molecules/ transcription factors/ modulators**			
Dual specificity phosphatase 6 (*DUSP6*)	1	2.1	∼
Ets variant gene 5 (*ETV5*)	1	2.0	−2.1
Ets variant gene 4 (*ETV4*)	1	1.8	−1.8
Heparin-binding EGF-like growth factor (*HBEGF*)	1	1.8	−1.5
Sprouty homolog 4 (*SPRY4*)	1	1.7	−2.7
G protein-coupled receptor kinase 5 (*GRK5*)	1	1.6	2.0
High mobility group AT-hook 2 (*HMGA2*)	1	1.6	−2.7
G protein-coupled receptor 153 (*GPR153*)	1	1.6	∼
Dual specificity phosphatase 4 (*DUSP4*)	1	1.6	−5.4
Leucine-rich repeats and immunoglobulin-like domains 1 (*LRIG1*)	1	1.5	∼
Follistatin, transcript variant FST317 (*FST*)	1	1.5	∼
Forkhead box A2 (*FOXA2*)	1	1.5	∼
**Cell cycle/ differentiation/ apoptosis**			
Immediate early response 3 (*IER3*)	1	1.6	−2.2
Tribbles homolog 2 (*TRIB2*)	1	1.6	∼
Pleckstrin homology-like domain, family A, member 1 (*PHLDA1*)	1	1.5	∼

Three KGFR-positive SCC cell lines were serum-starved and treated with rKGF (10 ng/ml) for 24 h. Total RNA was analyzed for differential gene expression by Affymetrix microarray. The genes with the hybridization signal above the median of the signal intensity distribution in at least one sample were analyzed. The genes with more than 1.5 fold (150% of control) change in the expression level were included in the table.

1 The data represents the fold-change of hybridization signal of KGF-treated cells over the untreated cells for the indicated gene.

2 The data represents the fold-change of hybridization signal between untreated SCC cells and normal epidermal keratinocytes for the indicated gene. The negative fold-change indicates decrease in hybridization signal in SCC cells compared to normal keratinocytes. ∼ indicates positive or negative fold-change less than 1.5.

KGF treatment downregulated the expression of 18 genes in cutaneous SCC cell lines. Interestingly, the expression of 13 of these downregulated genes was at least 2-fold higher in untreated SCC cells, as compared to normal epidermal keratinocytes (Table 2). These genes included several antiviral defense –related genes, genes encoding interferon-induced proteins, and genes for growth regulation and signaling. Among the most downregulated genes by KGF in SCC cells were four genes specifically up-regulated in SCC cells compared to normal keratinocytes: *MMP13* (matrix metalloproteinase-13, collagenase-3), *MATN2* (matrilin 2), *CXCL10* (chemokine (C-X-C motif) ligand 10, IP-10), and *IGFBP3* (insulin-like growth factor binding protein 3).

**Table 2:** Downregulation of different classes of genes in cutaneous SCC cells by KGF as determined by DNA microarray analysis.

Coded protein (*Gene name*)	Ctrl	rKGF^1^	SCC/normal^2^
**ECM/ECM receptors, ECM modulators, cytoskeleton**			
Matrix metallopeptidase 13 (*MMP13*)	1	−3.0	79.0
Matrilin 2 (*MATN2*)	1	−2.2	10.2
**Growth factors/ receptors/ signaling molecules/ transcription factors/ modulators/ chemokines**			
Chemokine (C-X-C motif) ligand 10 (*CXCL10*)	1	−2.6	36.6
Tumor necrosis factor ligand superfamily member 10 (*TNFSF10*)	1	−2.5	7.3
Insulin-like growth factor binding protein 3 (*IGFBP3*)	1	−2.4	5.6
Signal transducer and activator of transcription 2 (*STAT2*)	1	−2.0	2.4
**Antiviral defence/ interferon induced**			
Interferon-induced protein with tetratricopeptide repeats 1 (*IFIT1*)	1	−2.5	2.6
Radical S-adenosyl methionine domain containing 2 (*RSAD2*)	1	−2.2	7.5
Myxovirus (influenza virus) resistance 1, interferon-inducible protein p78 (mouse) (*MX1*)	1	−2.2	1.7
Tripartite motif-containing 22 (*TRIM22*)	1	−2.2	∼
Guanylate binding protein 1, interferon-inducible (*GBP1*)	1	−2.1	3.1
Interferon-induced protein with tetratricopeptide repeats 2 (*IFIT2*)	1	−2.0	24.0
2′,5′-oligoadenylate synthetase 1, 40/46 kDa (*OAS1*)	1	−2.0	1.6
**Micellaneous**			
Oxidized low density lipoprotein (lectin-like) receptor 1 (*OLR1*)	1	−2.8	12.7
Kynurenine 3-monooxygenase (*KMO*)	1	−2.4	6.1
StAR-related lipid transfer protein 5 (*STARD5*)	1	−2.1	1.9
Serum amyloid A1 (*SAA1*)	1	−2.0	∼
Solute carrier family 6 (neurotransmitter transporter, noradrenalin), member 2 (*SLC6A2*)	1	−2.0	4.0

Three KGFR-positive SCC cell lines were serum-starved and treated with rKGF (10 ng/ml) for 24 h. Total RNA was analyzed for differential gene expression by Affymetrix microarray. The genes with the hybridization signal above the median of the signal intensity distribution in at least one sample were analyzed. The genes with more than −2.0 fold (less than 50% of control) change in the expression level were included in the table.

1 The data represents the fold-change of hybridization signal of KGF-treated cells over the untreated control cells for the indicated gene. The negative fold-change indicates decreased hybridization signal compared to control.

2 The data represents the fold-change of hybridization signal between untreated SCC cells and normal epidermal keratinocytes for the indicated gene. ∼ indicates positive or negative fold-change less than 1.5.

**Figure 3:**
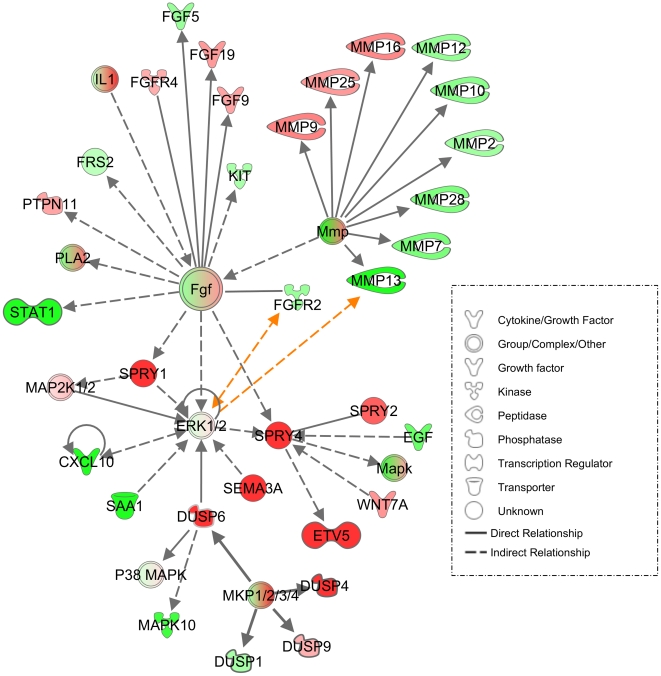
Dynamic molecular network induced by KGF in skin SCC cells. KGF-treatment of skin SCC cells induces up- and downregulation of variety of ERK1/2-regulated genes including upregulation of *DUSP6*, *ETV5*, *SPRY1*, and *SPRY4* genes. KGF also induces downregulation of *MMP7*, *MMP13* and *CXCL10*. The data presented show KGF-induced changes as an average in three SCC cell lines. The image was generated using Ingenuity Pathways Analysis (IPA). The symbols represent gene function, as indicated in legend on the right. Red color indicates gene upregulation and green color downregulation by KGF. The genes with fold change >1.5 are indicated with the strongest red (up) and green (down) colors. The rainbow color indicates a group of genes with up- or downregulation. Some of the groups (FGF, MMP, MKP1/3/4) are “opened” to show the regulation of individual genes. The arrows and lines indicate direct (solid line) and indirect (dashed line) physical and functional interactions. The arrows show the direction of regulation. The relations found in this study are highlighted with yellow arrows.

Analysis of KGF regulated genes in SCC cells with Ingenuity Pathway Analysis revealed functional relationship of several of these genes with ERK1/2 signaling pathway, including *DUSP6*, *SPRY4*, *CXCL10*, and *MMP-13* (Figure 3).

### The expression of matrilin 2, CXCL10, IGFBP3, DUSP4 and DUSP6 is regulated by KGF in SCC cells

**Figure 4:**
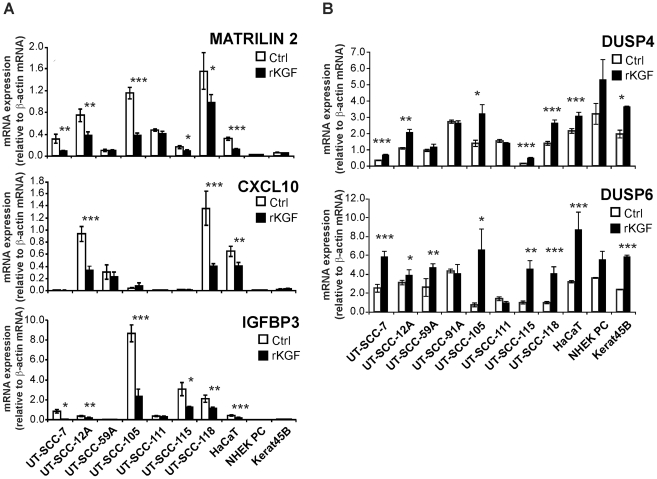
The expression of matrilin 2, CXCL10, IGFBP3, DUSP4 and DUSP6 is regulated by KGF in cutaneous SCC cells. Cutaneous SCC cell lines (UT-SCC-7, -12A, -59A, -91A, -105, -111, -115, and -118), HaCaT cells and normal keratinocytes (NHEK PC, Kerat45B) were serum starved, treated with recombinant KGF (rKGF; 10 ng/ml) for 24 h and analyzed for (**A**) matrilin 2, CXCL10 and IGFBP3 mRNA and (**B**) DUSP4 and DUSP6 mRNA expression with qPCR. The results were normalized for β-actin mRNA levels in each sample. Note that the cell lines UT-SCC-91A and UT-SCC-111 do not express KGFR mRNA. *p<0.05, **p<0.01, ***p<0.001, with independent samples T-test, n = 3–4.

Due to their association with extracellular matrix (ECM) homeostasis, regulation of angiogenesis and cancer progression and metastasis [31]–[35], the expression of matrilin 2, CXCL10, and IGFBP3 mRNA was further analyzed by qPCR in seven cutaneous SCC cell lines, including one KGFR negative cell line (UT-SCC-111), HaCaT cells and normal epidermal keratinocytes treated with rKGF (10 ng/ml) for 24 h. As expected, the expression of matrilin 2, CXCL10 and IGFBP3 mRNA was undetectable or very low in normal keratinocytes (Figure 4A). In accordance with the microarray data, the basal expression of matrilin 2 mRNA was markedly elevated in all SCC cell lines, as compared to normal keratinocytes, and was downregulated by KGF in 5 out of 6 KGFR positive SCC cell lines (Figure 4A, upper panel). CXCL10 mRNA expression was elevated in 3 out of 7 SCC cell lines compared to keratinocytes and KGF treatment downregulated the expression significantly in 2 SCC cell lines (Figure 4A, middle panel). The expression of IGFBP3 mRNA was detected in 6 out of 7 SCC cell lines, but not in normal keratinocytes (Figure 4A, lower panel). Downregulation of IGFBP3 mRNA levels by KGF was noted in 5 KGFR positive cell lines. As expected, KGF had no effect on the expression of any of the three genes in KGFR-negative cell line UT-SCC-111 (Figure 4A). In HaCaT cells the expression of matrilin 2, CXCL10, and IGFBP3 was clearly elevated, as compared to normal epidermal keratinocytes, and the expression of all three genes was significantly downregulated by KGF (Figure 4A).

The regulation of DUSP4 and DUSP6 expression by KGF was also verified by qPCR. The expression of DUSP4 mRNA was markedly upregulated in 5 out of 6 KGFR positive SCC cell lines and DUSP6 mRNA in all 6 KGFR positive SCC cell lines, as well as in normal keratinocytes and HaCaT cells (Figure 4B). DUSP4 and DUSP6 expression was not altered by KGF in KGFR negative SCC cell lines UT-SCC-91 and -111 (Figure 4B).

### KGF downregulates the expression of MMP-13 and MMP-7 and suppresses invasion of SCC cells

**Figure 5:**
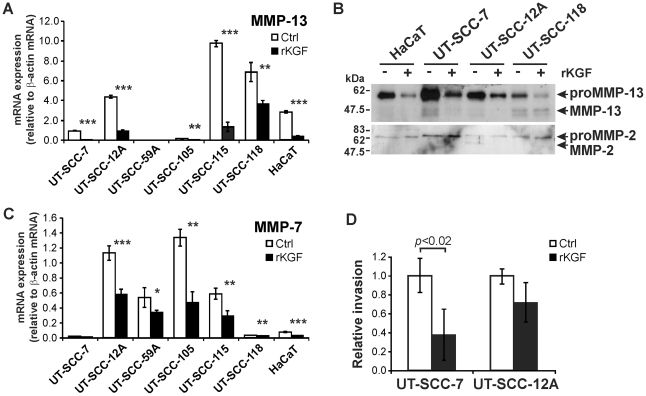
KGF downregulates the expression of MMP-13 and MMP-7 and suppresses invasion of cutaneous SCC cells. (**A, C)** Cutaneous SCC cell lines (UT-SCC-7, - 12A, -59A, -105, -111, and -118) and HaCaT cells were serum starved and treated with recombinant KGF (rKGF; 10 ng/ml). After 24-h treatment, total RNA was extracted and analyzed for the expression of MMP-13 (**A**) and MMP-7 (**C**) mRNA by qPCR. The results were normalized for β-actin mRNA levels in the same samples. *p<0.05, **p<0.01, ***p<0.0001, with independent samples T-test, n = 3–4. (**B**) HaCaT cells and cutaneous SCC cells (UT-SCC-7, - 12A, and -118) were incubated with recombinant KGF (rKGF) for 72 h, and the medium samples were analyzed for MMP-13 and MMP-2 protein using western immunoblotting. Each pair of control and KGF-treated sample contain equal amount of total protein. (**D**) Cutaneous SCC cells (UT-SCC-7, -12A) (3×10^5^cells) treated with KGF (10 ng/ml) for 24 h were applied in collagen gel coated invasion chamber and incubated for 48 h in the presence of KGF. The cells that had invaded through collagen matrix were stained, photographed and counted. Statistical analysis with independent samples T-test, n = 4–6.

Matrix metalloproteinase-13 (MMP-13) is a wide spectrum metalloendopeptidase implicated in invasion, vascularization, and growth of cutaneous SCC [31], [36]. In accordance with the microarray data, the expression of MMP-13 transcript was detected by qPCR in 5 out of 6 cutaneous SCC cell lines and also in HaCaT cells (Figure 5A). KGF treatment potently and significantly downregulated MMP-13 expression in all 5 SCC cell lines and in HaCaT cells (by 47–94%), as compared to corresponding untreated control cultures (Figure 5A). The analysis of the conditioned media of three SCC cell lines and HaCaT cells by western immunoblotting revealed a marked reduction in MMP-13 production after KGF treatment, as compared to corresponding untreated control cells (Figure 5B). In contrast, production of MMP-2 in the same cultures was unaltered by KGF.

MMP-7 has been identified as a marker for malignant transformation of epidermal keratinocytes in cutaneous SCCs [37], [38]. Analysis by qPCR revealed expression of MMP-7 transcript in 4 out of 6 cutaneous SCC cell lines and in HaCaT cells (Figure 5C) and KGF treatment resulted in a marked (34–65%) reduction in MMP-7 mRNA expression compared to untreated control cultures (Figure 5C). Furthermore, as shown in Figure 5D, downregulation of MMP-13 and MMP-7 expression by KGF was associated with reduction in invasion of UT-SCC-7 and -12A cells through collagen.

### KGF-elicited downregulation of MMP-13 expression by SCC cells is mediated via ERK1/2

**Figure 6:**
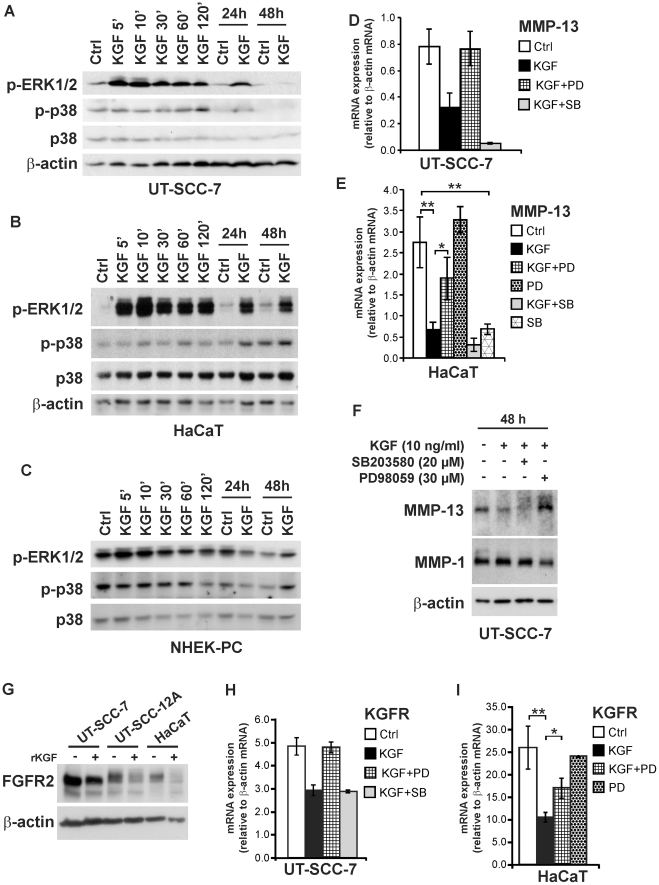
KGF-elicited downregulation of MMP-13 and KGFR expression in cutaneous SCC and HaCaT cells is mediated via ERK1/2. Serum-starved (**A**) skin SCC cells (UT-SCC-7), (**B**) HaCaT cells, and (**C**) human epidermal keratinocytes (NHEK-PC) were incubated with KGF (10 ng/ml) for different periods of time, as indicated. Cell lysates were analyzed for phosphorylated forms of ERK1/2 and p38 MAP-kinases (p-ERK1/2 and p-p38, respectively) and for total p38 by western immunoblotting. β-actin was determined as loading control. (**D, E**) Serum-starved (**D**) UT-SCC-7 cells (n = 2) and (**E**) HaCaT cells (n = 3) were pretreated with MEK1 inhibitor PD98059 (30 µM) or with p38 inhibitor SB203580 (20 µM) for 1 h and KGF (10 ng/ml) was added, as indicated. After 24 h, total RNA was harvested and analyzed for MMP-13 mRNA by qPCR. The results were normalized for β-actin mRNA levels in each sample. (**F**) UT-SCC-7 cells were treated as in (**D**) and incubated for 48 h. Equal aliquots of conditioned media were analyzed for MMP-13 and MMP-1 by western immunoblotting. The level of β-actin in corresponding cell lysstes was determined as loading control. (**G**) Serum-starved skin SCC cells (UT-SCC-7, -12A) and HaCaT cells were treated with recombinant KGF (10 ng/ml) for 24 h and equal amounts of total cell lysates were analyzed for FGFR2 by western immunoblotting. The expression level of β-actin was visualized for estimation of equal loading. (**H, I**) UT-SCC-7 and HaCaT cells were treated as in (**D, E**). After 24 h, total RNA was harvested and analyzed for KGFR mRNA by qPCR. The results were normalized for β-actin mRNA levels in each sample. *p<0.05, **p<0.01, with independent samples T-test, n = 3.

KGF has been reported to activate extracellular signal-regulated kinase 1/2 (ERK1/2) and p38 mitogen activated protein kinase (MAPK) in epithelial cells [39]–[42]. In addition, pathway analysis of genes regulated by KGF in SCC cells showed association of several of these genes with ERK1/2 signaling (Figure 3). As shown in Figure 6A and 6B, KGF induced rapid and persistent activation of ERK1/2 in UT-SCC-7 cells and in HaCaT cells, noted 5 min after addition of KGF and still persistent after 24 h (Figure 6A and B). After 48 h, activation of ERK1/2 had returned to control level in UT-SCC-7 cells, but was still prominent in HaCaT cells. In UT-SCC-7 cells, activation of p38 was detected after 2 h and 24 h and returned to control level in 48 h (Figure 6A). In HaCaT cells, activation of p38 was most potent after 24- and 48-h KGF treatment (Figure 6B). Rapid and transient activation of ERK1/2 was also noted in epidermal keratinocytes stimulated by KGF, but the activation was less potent as in SCC and HaCaT cells (Figure 6C).

To elucidate the role of ERK1/2 signaling pathway in mediating downregulation of MMP-13 by KGF, SCC cells were treated with PD98059, a small molecular inhibitor of ERK1/2 pathway. Parallel cultures were treated with SB203580, an inhibitor of p38 MAPK. Co-treatment of cells with PD98059 resulted in potent inhibition of the downregulatory effect of KGF on MMP-13 expression, whereas SB203580 potentiated the effect of KGF in UT-SCC-7 cells (Figure 6D). Similarly, the downregulatory effect of KGF on MMP-13 expression in HaCaT cells was abrogated by PD98059 and augmented by SB203580 (Figure 6E). PD98059 alone had no effect on MMP-13 expression (Figure 6E). In accordance with previous observations [43], SB203580 alone significantly inhibited the expression of MMP-13 mRNA (Figure 6E). Abrogation of KGF-induced downregulation of MMP-13 protein production by PD98059 in UT-SCC-7 cells was also evident, and SB203580 in combination with KGF markedly reduced the production of MMP-13 (Figure 6F). MMP-1 expression was unaltered by KGF treatment or by small molecule inhibitors (Figure 6F). The function of PD98059 and SB203580 was verified with immunoblotting for phospho-ERK1/2, and for phospho-Creb, downstream target of p38, respectively (data not shown).

Downregulation of KGFR (FGFR2-IIIb) expression after 24-h treatment with KGF was also noted at protein level by western immunoblotting of cell lysates of two SCC cell lines (Figure 6G). Downregulation of KGFR mRNA by KGF in SCC and HaCaT cells was also abrogated by PD98059 providing evidence for the role of ERK1/2 signaling in the regulation of KGFR expression (Figure 6H and 6I).

### KGF downregulates MMP-13 and KGFR expression in Ha-*ras*-transformed HaCaT cells *via* ERK1/2

**Figure 7:**
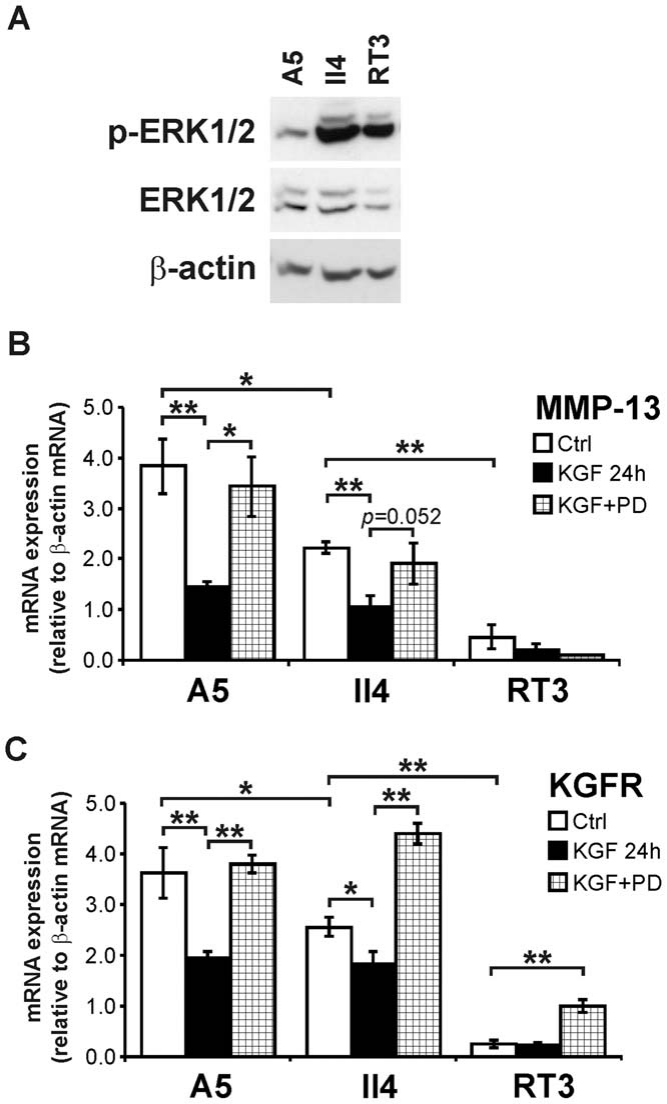
KGF downregulates MMP-13 and KGFR expression in Ha-*ras*-transformed HaCaT cells *via* ERK1/2. Three Ha-*ras*-transformed HaCaT cell-derived lines (A5, II4, and RT3) were used. A5 is benign tumorigenic cell line, II4 forms invasive malignant tumors, and RT3 cells form metastatic SCCs *in vivo*. (**A**) A5, II4, and RT3 cultures were serum-starved overnight and the cell lysates were analyzed for phosphorylated ERK1/2 (p-ERK1/2) and total ERK1/2 by western blotting. β-actin was determined as loading control. (**B, C**) Serum-starved A5, II4, and RT3 cells were pre-treated with PD98059 (30 µM) for 1 h and KGF (10 ng/ml) was added. After 24 h, total RNA was extracted and analyzed for MMP-13 mRNA (**B**) and KGFR mRNA (**C**) using qPCR. The amplification results were normalized for β-actin mRNA levels in each sample. *p<0.05, **p<0.005, with independent samples T-test, n = 3.

To further corroborate the role of ERK1/2 signaling in the downregulation of MMP-13 and KGFR in transformed epidermal cells, we examined MMP-13 expression in three Ha-*ras*-transformed HaCaT cell lines (A5, II4, and RT3), which represent an *in vitro* model for different stages of cutaneous SCC tumor progression. A5 is a benign tumorigenic cell line, II4 cells form invasive malignant tumors, and RT3 forms metastatic tumors *in vivo* [44], [45]. As shown in Figure 7A, invasive II4 cells and metastatic RT3 cells displayed more potent activation of ERK1/2, as compared to benign tumorigenic A5 cells. Interestingly, the expression level of MMP-13 mRNA correlated negatively with ERK1/2 activation and the tumorigenic potential of the cell lines (Figure 7B). In addition, the expression of KGFR mRNA correlated negatively with the tumorigenic potential of Ha-*ras*-HaCaT cells and their basal ERK1/2 activation (Figure 7C). KGF significantly downregulated MMP-13 expression and PD98059 inhibited the downregulation in A5 and II4 cells, but not in the metastatic cell line (RT3) which expressed KGFR and MMP-13 mRNA at very low level (Figure 7B,C). KGF also potently downregulated KGFR mRNA expression in A5 and II4 cells and co-treatment of cells with PD98059 potently abrogated KGF-induced downregulation of KGFR (Figure 7C).

### Activation of ERK1/2 downregulates MMP-13 and KGFR expression in HaCaT cells

**Figure 8:**
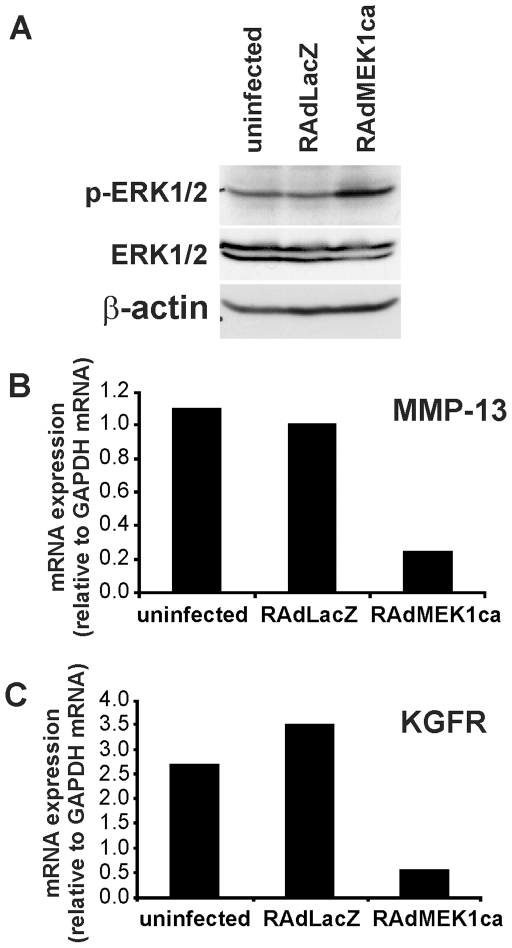
Activation of ERK1/2 downregulates MMP-13 and KGFR expression in HaCaT cells. Serum-starved HaCaT cells were infected with control adenovirus RAdLacZ or with adenovirus RAdMEK1ca harboring constitutively active MEK1 at MOI 500. (**A**) Cell lysates were harvested 18 h after infection and analyzed for phosphorylated ERK1/2 (p-ERK1/2) and total ERK1/2 by western blotting. β-actin was determined as loading control. (**B**) Total RNA was extracted 48 h after infection and analyzed for MMP-13 mRNA by qPCR. The amplification results were normalized for GAPDH mRNA levels in each sample. (**C**) Total RNA was extracted 24 h after infection and analyzed for KGFR by qPCR, as described in (**B**).

To study the specific role of ERK1/2 pathway in the downregulation of MMP-13 and KGFR expression, we used adenoviral gene delivery (RAdMEK1CA) of constitutively active MEK1 (the upstream kinase of ERK1/2) to obtain specific activation of ERK1/2 (Figure 8A). As shown in Figures 8B and 8C, activation of ERK1/2 in HaCaT cells resulted in marked reduction in MMP-13 and KGFR mRNA expression, as compared to cells transduced with control adenovirus (RAdLacZ). Together these results (Figures 7 and 8) provide evidence for the involvement of ERK1/2 signaling in downregulation of MMP-13 and KGFR expression in SCC cells and show, that specific and sustained ERK1/2 activation results in downregulation of MMP-13 and KGFR expression.

## Discussion

In this study we have examined the effect of KGF, a potent epithelial cell mitogen, on cutaneous SCC cells. Our results show, that most SCCs of skin *in vivo* express mRNAs for KGF and KGFR, and that the level of KGFR expression is significantly lower than in normal skin. The expression of KGFR was also detected in the majority of primary and metastatic cutaneous SCC cell lines, although 20% of the cell lines were negative for KGFR. Interestingly, treatment of KGFR positive SCC cell lines with recombinant KGF or by conditioned medium of human skin fibroblasts overexpressing KGF after recombinant adenoviral infection did not stimulate SCC cell proliferation indicating altered response to KGF. In contrast, KGF clearly stimulated proliferation of HaCaT cells and normal epidermal keratinocytes. KGF treatment of cutaneous SCC cells induced a rapid and persistent activation of ERK1/2, which was over by 48 h, whereas in HaCaT cells ERK1/2 activation induced by KGF was more persistent and persisted at least 48 h. In normal keratinocytes the activation of ERK1/2 was rapid and transient. Thus, although ERK1/2 was rapidly activated in both SCC cells and HaCaT cells the results of the present study do not provide mechanistic explanation for the difference between SCC cells and HaCaT cells with respect to the mitogenic response to KGF. This difference could be related to the magnitude and persistence of ERK1/2 activation by KGF, which appeared less prominent in SCC cells compared to HaCaT cells. However, our results show, that in cutaneous SCC cells, KGF induced activation of ERK1/2 does not mediate mitogenic signaling, in contrast to HaCaT cells and normal epidermal keratinocytes. As HaCaT cells, which harbor inactivation of both p53 alleles represent an early step in the epidermal carcinogenesis, it is possible that KGF stimulates proliferation of p53 negative keratinocytes in the UV-damaged skin. However, as a result of malignant transformation, keratinocytes appear to lose the mitogenic response to KGF, and also KGFR expression as will be discussed below. These observations are in accordance with previous findings on lack of mitogenic effect of KGF on head and neck SCC cells [24].

KGF was found to downregulate the expression of KGFR mRNA in cutaneous SCC cells as well as in HaCaT cells indicating functional negative feedback regulation of KGFR signaling. It was also noted that in Ha-*ras*-transformed HaCaT cell lines, activation of ERK1/2 negatively correlates with the expression level of KGFR mRNA and inhibition of ERK1/2 signaling restores KGFR mRNA expression, demonstrating ERK1/2 dependent negative regulation of KGFR expression. Moreover, the expression level of KGFR decreased with the increasing tumorigenic potential of Ha-*ras*-HaCaT cells. Thus, it is likely, that the constitutive activation of ERK1/2 in a subset of the invasive and metastatic cutaneous SCCs results in reduced response to KGF *via* downregulation of KGFR expression. In addition, skin SCC tumors showed reduced levels of KGFR expression compared to normal skin *in vivo*, and a subset of cutaneous SCC tumor cell lines were negative for KGFR. Together these results suggest that the expression of KGFR is downregulated and lost upon malignant transformation of keratinocytes and during their progression to invasive and metastatic cutaneous SCC cells. This is consistent with the reports on the expression pattern of KGFR detected in esophageal, bladder, and hepatocellular carcinoma [20], [21], [46].

Dissection of KGF response in skin SCC cells by microarray based gene expression profiling and pathway analysis demonstrated upregulation of several known targets genes for ERK1/2 signaling, including *ETV4* and *ETV5*, which code for Ets transcription factors ETV4 and ETV5, respectively, and MAPK phosphatases *DUSP4* and *DUSP6*. DUSP6 is inducible by FGF signaling, it is transcriptionally regulated by Ets protein, and it specifically inactivates ERK1/2 [47], [48]. Additional ERK1/2-activated genes induced by KGF-treatment included *IER3*, which has been shown to stimulate proliferation of HaCaT cells under normal conditions, but sensitize them to stress-induced apoptosis [49], proapoptotic *PHLDA1* [30] and *SPRY4* (Sprouty 4), which belongs to a family of receptor tyrosine kinase inhibitors which can inhibit ERK1/2 activation [50]. Of these, DUSP4 and DUSP6, PHLDA1 and Sprouty 4 are characterized by tumor suppressing properties [26]–[28], [30]. Moreover, *LRIG1*, a gene coding for a protein with recognized tumor suppressing properties, was upregulated by KGF in SCC cells. LRIG1 plays a role in skin homeostasis as a negative feedback regulator of EGF signaling by inducing ubiquitination and degradation of EGFR [29]. Thus, several genes that may play a role in the negative feedback regulation of KGFR signaling were upregulated by KGF in cutaneous SCC cells. In addition, induction of genes involved in the regulation of ERK1/2 activity could also partially explain the uncoupling of ERK1/2 activation from mitogenic signaling of KGF response in SCC cells.

We also identified three novel KGF-regulated genes, *MATN2*, *CXCL10*, and *IGFBP3*, which are upregulated in skin SCC cells in comparison with epidermal keratinocytes and potently downregulated by KGF. Matrilin 2 (MATN2) is a putative adaptor protein for ECM assembly, which is widely expressed and localizes especially to the basement membrane zones in skin and blood vessels in normal and malignant tissues [51]–[53]. Chemokine CXCL10 is a ligand for chemokine receptor CXCR3, and its expression is upregulated in head and neck SCCs [54]. CXCL10 has been shown to promote invasion-related properties of colorectal carcinoma cells [55], and it also regulates angiogenesis [32]. IGFBP3 stabilizes insulin-like growth factors (IGFs) affecting their affinity to IGF receptors, this way serving as a negative regulator of cell proliferation and as a promoter of apoptosis [33], [56]. The expression of IGFBP3 is upregulated in oral SCC tumors and cell lines [35] and in breast cancer, in which the expression of IGFBP3 is associated with poor outcome [57]. It is likely, that downregulation of the expression of these genes in cutaneous SCC cells by KGF modulates biological processes important in progression of cutaneous SCCs, such as cell migration and proliferation, inflammation, angiogenesis and ECM assembly.

*MMP13* (collagenase-3) was identified as the most potently downregulated gene by KGF in the cutaneous SCC cells. MMP-13 is not expressed by normal epidermal keratinocytes in intact skin or during wound repair, but it is readily expressed by tumor cells in cutaneous SCCs [37], [38], [58]. MMP-13 can cleave a wide range of ECM and non-matrix molecules [59] and it has been implicated in invasion, growth, and vascularization of cutaneous SCCs [31], [36]. Our results also show, that KGF does not downregulate MMP-13 expression, when activation of ERK1/2 is abolished by PD98059, indicating that ERK1/2-signaling is required for KGF-induced downregulation of MMP-13 expression. This is in accordance with previous observation showing that IFN-γ reduces MMP-13 expression in cutaneous SCC cells in ERK1/2-dependent manner [60]. In addition, constitutive activation of ERK1/2 either by oncogenic Ha-*ras* or by constitutively active MEK1 resulted in marked downregulation of MMP-13 expression in epidermal keratinocyte-derived HaCaT cells harboring p53 inactivation. This supports the hypothesis that constitutive activation of ERK1/2 results in downregulation of MMP-13 expression. It is also interesting to note that KGF potently downregulates MMP-13 expression in non-tumorigenic (HaCaT) and benign tumorigenic (A5) keratinocyte-derived cell lines, which are phenotypically similar to epidermal keratinocytes in UV-induced premalignant SCC precursor lesions (actinic keratoses). These results suggest that KGF does not promote progression of premalignant actinic keratoses to invasive SCCs.

*MMP7* (matrilysin-1) was also identified as a novel target gene for KGF in skin SCC cells. MMP-7 is not expressed by keratinocytes in normal epidermis or in cutaneous wounds, but it is expressed by tumor cells in cutaneous SCCs [37], [38]. MMP-7 is capable of cleaving various ECM components, including collagen type IV, fibronectin, laminins and proteoglycans [61], [62]. MMP-7 can also regulate cell adhesion and migration by shedding syndecan-1 and E-cadherin, and promote cell proliferation by activating HB-EGF from cell surface [63]–[65]. Thus, downregulation of two transformation-specific MMPs, MMP-13 and MMP-7, in cutaneous SCC cells by KGF provides a mechanistic explanation for inhibition of SCC cell invasion.

In conclusion, the results of the present study show that although most cutaneous SCC cell lines express KGFR, they are unresponsive to the mitogenic effect of KGF. Furthermore, the expression of KGFR mRNA was found to correlate negatively with tumorigenic potential of Ha-*ras*-transformed HaCaT cells. KGF reduced invasion capacity of KGFR-positive cutaneous SCC cells and induced a specific gene expression signature characterized by upregulation of a panel of genes associated with tumor suppression and downregulation of several genes linked to tumor progression. These results provide evidence for a role for KGF as a suppressor of malignant phenotype of skin SCC cells. Based on these findings, we propose that KGF does not promote progression of cutaneous SCCs, but rather suppresses the malignant phenotype of SCC cells. However, this beneficial effect of KGF appears to be compromised in the most aggressive SCC cells due to downregulation of KGFR expression by a mechanism involving activation of ERK1/2 signaling pathway and in a subset of cells, by eventual loss of KGFR.

## Materials and Methods

### Ethics statement

All studies involving human patients were approved by the Joint Ethical Committee of the University of Turku and Turku University Hospital. Participants gave their informed consent in writing, and the study was conducted according to declaration of Helsinki.

### Tissue samples

Cutaneous SCC tumor samples (n = 6) were collected from surgically removed primary tumors in Turku University Hospital. Normal skin samples were obtained from patients undergoing mammoplasty (n = 6) in Turku University Hospital.

### Cell cultures

Cutaneous SCC cell lines (n = 8) were established at the time of operation from five primary (UT-SCC-12A, -91, -105, -111 -118) and three metastatic tumors (UT-SCC-7, -59A, -115) in Turku University Hospital [66]. SCC cells were cultured in DMEM supplemented with penicillin and streptomycin, 2 mM L-glutamine, nonessential amino acids and 10% fetal calf serum (FCS). HaCaT cells [25] and normal human skin fibroblasts [67] were cultured in DMEM containing antibiotics, L-glutamine and 10% FCS. Ha-*ras*-transformed HaCaT cells (A5, II4, RT3) [44], [45] were cultured in DMEM containing G418 (200 µg/ml), antibiotics, L-glutamine and 10% FCS. HaCaT cells and Ha-*ras*-transformed HaCaT cells were kindly provided by Dr. Norbert E. Fusenig, (German Cancer Research Center, Heidelberg, Germany). Normal epidermal keratinocytes were established from normal skin of patients undergoing mammoplasty [66] or purchased from PromoCell Gmbh (Heidelberg, Germany). Keratinocytes were cultured in Keratinocyte Growth Medium −2 supplemented with SupplementMix (PromoCell).

### Cell treatment with recombinant KGF and MAPK inhibitors

Cutaneous SCC cells or HaCaT cells were serum-starved overnight and incubated with bacterial recombinant human KGF (K1757; Sigma-Adrich, St. Luis, MO, USA) for indicated periods. Keratinocytes were cultured in normal growth medium, and subsequently treated in a similar manner. Inhibitors of MEK1/2 (PD98059; Calbiochem, San Diego, CA) and p38 MAPK (SB203580; Calbiochem, San Diego, CA) were added in 30 µM and 20 µM concentrations, respectively, one hour prior to KGF.

### Construction of KGF adenovirus and adenoviral gene delivery

Recombinant replication-deficient adenovirus harboring KGF cDNA was constructed as described previously [67]. Human KGF coding cDNA was amplified from dermal fibroblast total RNA and cloned into pCA3 shuttle vector (Microbix Biosystems, Toronto, ON, Canada). pCA3 construct and pBHG10 plasmid containing adenovirus genome were co-transfected into HEK293 cells (Microbix Biosystems, Toronto, ON, Canada). One KGF-positive adenovirus clone was selected for the production of high titer preparation. Human dermal fibroblasts were infected with RAdKGF or RAdpCA3 control vector in suspension at MOI 200 and plated [67]. The media were changed next day and harvested 72 h later. KGF concentration in the media were determined by ELISA, Quantikine Immunoassay, human KGF (DKG00, R&D Systems, Minneapolis, MN, USA). Recombinant replication-deficient adenovirus RAdLacZ (kindly provided by Gavin W. G. Wilkinson) used as a control virus and RAdMEK1ca harboring constitutively active mutant of MEK1 (kindly provided by Marco Foschi, University of Florence, Florence, Italy) have been described before [68], [69]. HaCaT cells were cultured in 0.5% FCS DMEM overnight and infected with adenovirus at multiplicity of infection (MOI) 500 for 6 h.

### RNA analysis by RT-PCR and Real-Time Quantitative RT-PCR

Total RNA was harvested from cells using Qiagen RNeasy kit (Qiagen GmbH, Hilden, Germany) and 1 µg was DNase-treated and reverse trancribed to cDNA using random oligohexamer primers. For KGFR, cDNA was ampified using PCR-primers (frw5′-CACTCGGGGATAAATAGTTC-3′ and rev5′-CTTGCTGTTTTGGCAGGACA-3′) designed to recognize the specific IIIb-type exon of FGFR2-transcripts [3] generating in a nucleotide fragment of 148 bp. The forward primer 5′-CCCCTTCATTGACCTCAACT-3′ and reverse primer 5′-ATGACCTTGCCCACAGCCTT-3′ were used to amplify a 550 bp fragment of glyceraldehyde-3-phosphate-dehydrogenase (GAPDH) transcript as loading control. Equal aliquots of each reaction were run in agarose gel containing ethidiumbromide to visualize DNA fragments.

Quantitative analysis of mRNA expression was performed from 3 or 4 laboratory replicates of untreated and KGF-treated (10 ng/ml, 24 h) cells [66]. 4 ng aliquots of cDNA were used in each reaction run in three experimental replicates. All mRNAs were normalized against amplification of house-keeping genes β-actin or GAPDH. Standard deviation of the experimental replicates in one run was ≤3% of the Ct mean for each sample. Tissue RNAs were extracted and processed for qPCR and run in two experimental replicates, as described previously [66]. Sequences for specific primers and probe for MMP-13 have been published [66]. In addition, the sequences of the gene specific primers and probes used are presented in Table 3.

**Table 3:** Sequences of primers and probes used for quantitative real-time RT-PCR.

β-actin	forward	5′-TCACCCACACTGTGCCCATCTACGC-3′
	reverse	5′-CAGCGGAACCGCTCATTGCCAATGG-3′
	probe	FAM-5′-ATGCCCTCCCCCATGCCATCCTGCGT-3′-TAMRA
KGFR	forward	5′-CCCTACCTCAAGGTTCTC-3′
	reverse	5′-TCGGTCACATTGAACAG-3′
	probe	FAM-5′-TAAATAGTTCCAATGCAGAAGTGCTGGC-3′-TAMRA
KGF	forward	5′-AGGGACCCAAGAGATGAAG-3′
	reverse	5′-TGATTGCCACAATTCCA-3′
	probe	FAM-5′-TATCATGGAAATCAGGACAGTGGCAGT′-3′-TAMRA
MMP-7	forward	5′-GTTAAACTCCCGCGTCATAGAAA-3′
	reverse	5′-TTGGAAAGAGTGAGTATTCTCCAACA-3′
	probe	FAM-5′-CAGAAGCCCAGATGTGGAGTGCCAG-3′-TAMRA
Matrilin2	forward	5′-TGGCTGCGAACACATTTGTGT-3′
	reverse	5′-TTGGGCCTTCAGTGCATTTCTTG-3′
	probe	FAM-5′-AATGGGAATTCCTACATCTGCAAATGC-3′-BHQ
CXCL10	forward	5′-GTTAATCCAAGGTCTTTAGA-3′
	reverse	5′-TGATCTCAACACGTGGACAAA-3′
	probe	FAM-5′-ACTTGAAATTATTCCTGCAAGCC-3′-BHQ
IGFBP3	forward	5′-TCTCAGAGCACAGATACCCAGAACTTC-3′
	reverse	5′-ACGGCAGGGACCATATTCTG-3′
	probe	FAM-5′-TCTCAGAGCACAGATACCCAGAACTTC-3′-BHQ
DUSP4	forward	5′-TCACGGCTCTGTTGAATGTCT-3′
	reverse	5′-TGTCGGCCTTGTGGTTATCTTC-3′
	probe	FAM-5′-CCTCGGACTGCCCAAACCACTT-3′-BHQ
DUSP6	forward	5′-GCCGCAGGAGCTATACGAG-3′
	reverse	5′-ACCGGCAGGTTACCCTTCT-3′
	probe	FAM-5′-TCGTCGCACATCGAGTCGGC-3′-BHQ

### Microarray analysis of gene expression profiling

Genome wide gene expression profiling was performed at The Finnish DNA Microarray and Sequencing Centre, Turku, Finland. 100 ng of total RNA was processed for hybridization to Affymetrix Human Gene 1.0 ST Array [70]. CEL-files were extracted with GCOS Manager 1.4. The data quality was checked using Affymetrix Expression Console™ software, and for probe set level intensity comparisons, Expression Console™ and RMA algorithm were used to generate CHP array result files from CEL-files. All microarray data is MIAME compliant and has been deposited in the public database GEO (Gene Expression Omnibus, NCBI; accession number GSE34652). The genes with more than −2.0 fold (≤50%) or 1.5 fold (150%≤) changes in expression level between the KGF-treated sample and control sample were considered to be differently regulated. The data presented are ratios of hybridization signal between treated and untreated control cell samples. Only the genes with the signal above the median of the signal intensity distribution present in at least one sample were included. Ingenuity Pathway Analysis (Ingenuity Systems, www.ingenuity.com) was employed to visualize molecular interaction networks induced by KGF in skin SCC cell lines.

### Immunoblotting

Equal aliquots of cell culture media or cell lysates were analyzed by Western immunoblotting, as previously described [66]. Following antibodies were used: mouse monoclonal antibodies against human MMP-13 (IM64L; Millipore, Billerica, MA) and β-actin (A1978; Sigma-Adrich, St. Louis, MO, USA); rabbit polyclonal antibodies against human FGFR2 (F0300, Sigma-Adrich, St. Louis, MO, USA), MMP-2 (AB809; Chemicon International Inc., Temecula, CA), MMP-1 (AB8105; Millipore, Billerica, MA), total and phosphorylated p38 (#9212 and #9211, respectively), and ERK1/2 (#9102 and #9101, respectively), and phosphorylated Creb (#9191) (Cell Signaling Technology, Beverly, MA) and goat polyclonal anti-human FGF-7 (C-terminal peptide) (C-19) (sc-1365, Santa Cruz Biotechnology, Inc., Santa Cruz CA).

### Cell Invasion Assay

Cell culture chambers with 8 µm pore size (BD Falcon, Franklin Lakes, NJ) were pre-coated with 1 mm thick layer of neutralized bovine collagen (2.1 mg/ml, PureCol, Advanced BioMatrix, Tucson, AZ). Cells were serum starved and treated with KGF (10 ng/ml) for 24 h, suspended (3×10^5^ cells) in 300 µl DMEM containing 0.1% BSA and applied on top of gels. DMEM containing 10% FCS was used as chemoattractant in the lower chamber. The upper and the lower chambers contained KGF. After 48 h, the gels including non-invaded cells were removed inside the upper chamber and the cells that had migrated through the pores to the other side of the chamber bottom, were stained with Hoechst 33342 and counted in comparable areas [66].

### Cell proliferation assays

DNA-synthesis was measured with colorimetric immunoassay quantifying the incorporation of 5-bromo-2-deoxyuridine (BrdU) into DNA, as described previously [71]. BrdU was added in culture media 2 h after starting the growth factor stimulation and incubated for 18 h. For cell stimulation by conditioned cell culture medium, BrdU was added 18 h after starting the stimulation and incubated for 8 h.

Relative number of viable cells in culture was assayed using colorimetric assay quantifying metabolism of WST-1 reagent according to manufacturer's instructions (Roche Applied Science, Mannheim, Germany). Cells were serum starved overnight and incubated with rKGF for 48 h before addition of WST-1.

### Statistical analysis

Data obtained from qPCR and invasion assays were analyzed by 2-sided two independent samples T-test utilizing Levene's test for equality of variances. Data from proliferation assays comparing control to each treatment were analyzed using 2-sided two independent samples Mann-Whitney test. All tests were conducted with SPSS 16.0 software (SPSS Inc.). *P*-values<0.05 were considered statistically significant. All the experiments were repeated at least two times or separately with different cell lines as indicated.
